# Effect of Inoculation with Autochthonous Lactic Acid Bacteria on Flavor, Texture, and Color Formation of Dry Sausages with NaCl Partly Substituted by KCl

**DOI:** 10.3390/foods13111747

**Published:** 2024-06-02

**Authors:** Jiawang Wang, Jiasheng Lu, Xin Zhang, Baohua Kong, Yongjie Li, Qian Chen, Rongxin Wen

**Affiliations:** 1 College of Food Science, Northeast Agricultural University, Harbin 150030, China; wangjiawang2y@126.com (J.W.); neau_lujiasheng@163.com (J.L.); 18846066085@163.com (X.Z.); kongbh63@hotmail.com (B.K.); liyongjie25@163.com (Y.L.); 2 College of Life Sciences, Yantai University, Yantai 264005, China

**Keywords:** low-sodium dry sausage, NaCl substitutes, autochthonous starter culture, quality, flavor characteristics

## Abstract

The effects of inoculating lactic acid bacteria (LAB), specifically *Lactiplantibacillus plantarum*, *Latilactobacillus sakei*, *Latilactobacillus curvatus*, and *Weissella hellenica* on the flavor, texture, and color formation of dry sausages in which NaCl was partially substituted by 40% KCl, were explored in this study. It was found that LAB inoculation increased the presence of ketones, alcohols, acids, esters, and terpenes. It also reduced the pH, moisture, protein, and fat content, improving the b*-value, flavor, and texture of the sausages. Notably, *L. sakei* inoculation showed the most significant improvement in dry sausages with NaCl substitutes, especially on the reduction of bitterness. Meanwhile, there was a close positive correlation between the LAB count with the alcohols and esters formation of dry sausage with NaCl substitution (*p* < 0.05). These findings offer insight into improving the product characteristics of dry sausages using NaCl substitutes.

## 1. Introduction

Traditional dry sausage is one of the popular fermented meat products in the northeast of China, and this sausage is usually made from pork back fat, lean pork, salt (NaCl), sugar, wine, nitrite, and a blend of spices to ferment naturally for approximately 10 days [1]. NaCl is a salt, enhancing the texture of fermented meat products, fostering flavor development, and inhibiting the growth of spoilage microorganisms. As fermentation progresses, the NaCl content typically rises to approximately 6.0% due to dehydration [2]. High sodium consumption is linked to various cardiovascular and cerebrovascular health risks, including heart and hypertensive diseases [3]. Fermented meats, a part of the human diet, are an important source of dietary salt [1]. Consequently, there is an urgent need to lower the sodium content in traditional fermented meats without compromising quality, aligning with the “low-salt” health-conscious trend.

Current sodium-reduction strategies for meat products encompass direct NaCl reduction, its substitution with other chloride salts, and the synergistic addition of flavor enhancers, among others [4]. While directly decreasing NaCl is straightforward, it can negatively impact product quality and limit the extent of reduction. Replacing NaCl with alternatives like KCl, MgCl_2_, CaCl_2_, and ZnCl_2_ is prevalent. However, when the substitution exceeds 40%, adverse effects on flavor (e.g., bitterness and metallic tastes) and texture emerge [5]. To address these flavor gaps, flavor enhancers—organic acids, amino acids, flavored peptides, and yeast extracts—are often integrated [6,7]. However, this method not only increases production costs but also deviates from “clean label” trends.

Using starter cultures as an inoculant has emerged as a promising approach to improve the quality of low-sodium fermented meats. These cultures not only promote flavor development and color improvement but also inhibit the growth of spoilage microorganisms that can arise from sodium reduction [8]. Lactic acid bacteria (LAB) are a prevalent starter culture in dry sausages [9]. They show a certain protein- and lipid-decomposition activity, which can increase the production of flavor compounds by metabolizing proteins and lipids. Meanwhile, bacteriocin and other bacteriostatic substances can be produced to combat the proliferation of harmful microorganisms caused by the reduction of sodium and decrease the build-up of harmful substances [10].

A total of 37 autochthonous LAB were screened from naturally fermented sausages in a previous study. Among them, *Lactiplantibacillus plantarum*, *Latilactobacillus sakei*, *Latilactobacillus curvatus*, and *Weissella hellenica* demonstrated superior fermentation abilities, and notably enhanced the flavor and sensory qualities of sausages with direct sodium reduction [9]. Additionally, our earlier findings showed that replacing NaCl with 40% KCl adversely affected the flavor and sensory qualities of sausages [11]. Hence, we hypothesized that these LAB strains might also address the flavor and sensory shortcomings in dry sausages with a 40% NaCl substitution.

In this study, we examined the influence of *L. plantarum*, *L. curvatus*, *L. sakei*, and *W. hellenica* on the texture, color, and flavor profiles of dry sausages, where NaCl was partially replaced by 40% KCl. A correlation analysis was employed to discern the associations of physicochemical aspects, quality, and flavor traits of these modified dry sausages throughout fermentation. This investigation aims to devise a method for refining low-sodium dry sausages and thereby further reduce sodium intake.

## 2. Materials and Methods

### 2.1. Starter Culture Preparation

From our previous research, *L. plantarum*, *L. sakei*, *L. curvatus*, and *W. hellenica* were selected as starters due to their demonstrated good antimicrobial activity and fermentation ability [9]. A de Man, Rogosa, and Sharpe (MRS) broth was used to propagate these LAB at 37 °C for 18 h. Following cultivation, the cultures were collected after 10 min centrifugation at 10,000× *g*. The cultures were washed with sterile water before use, as per the procedure outlined by Montanari et al. [12].

### 2.2. Dry Sausage Manufacture

Three distinct batches were prepared for each treatment on different days, following the procedure of Hu et al. [1]. The ingredients were as follows: 3600 g of lean pork and 400 g of pork back fat was chopped and mixed with dextrose (100 g), *Daqu* liquor (a rich aromatic Chinese liquor) (20.0 g), gourmet powder (6.0 g), spice powder (6.0 g), sodium ascorbate (0.20 g), and sodium nitrite (0.18 g). The spice powder contains aniseed, *Fructus amomi*, *Angelica sinensis*, cinnamon, *Syzygium aromaticum*, cumin, *Pericarpium zanthoxyli*, pepper powder, and *Amomum kravanh*.

The six treatments of dry sausage were as follows: The traditional sausages with 2.50% NaCl (CT) and sausages using NaCl substitutes with 1.50% NaCl and 1.00% KCl (CS, equal to replacing NaCl with 40% KCl) were used as controls. Other sausages using NaCl substitutes were inoculated (10^7^ CFU/g of meat) with *L. plantarum*, *L. sakei*, *L. curvatus*, and *W. hellenica*, respectively. These were respectively named LC, LS, WH, and LP. The mixed meat batter was stuffed into swine casings, and each sausage weighed approximately 0.15 kg and had a diameter of approximately 3.0 cm.

The fermentation parameters were as follows: A total of 150 sausages (6 treatments × 25 sausages [each treatment]) were manufactured for each batch. At 25 ± 2 °C, the sausages were dried (30−50% relative humidity) for 1 day and fermented (65−70% relative humidity) for 8 days. On days 0, 3, and 6 of fermentation, 54 sausages (6 treatments × 3 sausages [each treatment] × 3 sampling points) for each batch were used for determining the pH, moisture, protein, fat content, LAB count, color, and shear force. On day 9 of fermentation, 72 sausages (6 treatments × 12 sausages [each treatment]) for each batch were measured not only for the above indicators but also for the evaluation of sensory properties, volatile compounds, and the electronic nose (E-nose).

### 2.3. Determination of Chemical Composition, pH, and Lactic Acid Bacteria Count

For the chemical composition, the moisture content was measured by reference to the procedures of the Association of Official Analytical Chemists [13]. The protein and fat content were analyzed based on the ISO standards [14,15]. The pH was analyzed by reference to the procedures of Aro et al. [16]. For this, sausages (10.0 g) were homogenized with sterile water (100.0 mL), and the resultant mixture’s pH was analyzed with a pH meter (Mettler Toledo, Shanghai, China). At 37 °C, the plate-counting method was used to determine the LAB count, which were incubated for 48 h on MRS agar [1].

### 2.4. Determination of Color and Shear Force

The color attributes of the dry sausages, specifically lightness (L*), redness (a*), and yellowness (b*), were measured using a colorimeter (Juki Corp., Tokyo, Japan). To prepare for measurement, 3.0 g of the dry sausages was finely chopped and uniformly spread across the bottom of the colorimeter cup. A Texture Analyzer (Stable Micro Systems, Surrey, UK) with a shear blade (Warner-Bratzler G146, CA, USA) was used for measuring shear forces. The sausages were steamed at 90 °C for 20 min. Following steaming, they were allowed to cool and then sliced into 5.0 cm × 1.5 cm × 1.5 cm strips, and the test speed was 2.0 m/s [17].

### 2.5. Electronic Nose Analysis

An E-nose system (Schwerin, Germany) fitted with ten metal sensors was used to analyze the odor profiles of the sausages, using ten sensors as mentioned by Shi et al. [18]. To prepare for the assessment, 3.0 g of the dry sausages was chopped and placed in a headspace vial (CNW Technologies, Duesseldorf, Germany). The sample was heated for 30 min to release the odors at 40 °C. The E-nose system’s measurement duration for each sample was set to 90 s.

### 2.6. Volatile Compound Analysis

According to the description of Jin et al. [19], employing aheadspace solid-phase microextraction and gas chromatography–mass spectrometry (HS-SPME-GC-MS) on a GC-MS instrument from Shimadzu Corporation, Kyoto, Japan, for analysis. The procedures of Bicchi [20] and Jelen et al. [21] were referenced to correctly identify volatile compounds. To prepare the sample, 3.0 g of dry sausages and 4.0 μL of an internal standard (100 mg/L of 1,2-dichlorobenzene) were introduced into a headspace vial. This vial was sealed with a polytetrafluoroethylene septum. After sealing, the vial was balanced at 50 °C for 20 min. The volatile compounds were then extracted with SPME for 40 min, which employed a divinylbenzene/carboxen/polydimethylsiloxane fiber. The volatile compounds were screened by comparing the mass spectrometry with the NIST20 library and calculating them against the retention index of n-alkanes ranging from C7 to C40.

### 2.7. Sensory Evaluation

A quantitative descriptive was used to analyze the sensory properties on day 9 of fermentation [9]. The approval of the Research Ethics Committee is not usually required to conduct sensory evaluations at our institutions. There were not any human or animal experiments in this study. Participants were instructed to spit out the samples after analysis, and the samples were common and safe, so ethics committee approval was not required. The evaluation panel comprised 20 panelists with an age of 20–30 years (10 men and 10 women), all of whom possessed experience in the sensory analysis of dry sausage. Training and evaluation adhered to the standards set by ISO 8586 and ISO 8589-1 [22,23]. Three training sessions were held in two weeks. The initial two sessions were dedicated to establishing descriptive terms for evaluating sausages. To reach a consensus, panelists referred to samples associated with debate descriptors in the third session. The sensory characteristics were analyzed from 1 (not perceived) to 7 (maximum perception). A total of 108 sausages (6 treatments × 6 sausages [each treatment] × 3 batches) were randomly evaluated in 6 sessions, of which 18 sausages (6 treatments × 3 sausages [each treatment]) were evaluated in a session/day. The mean scores for each session and from all the panelists were calculated for statistical analysis. In order to maintain good safety and quality, dry sausage usually needs to be cooked and eaten in China, so sausages selected at random were first heated at 90 °C for 20 min, then cooled before being sliced and randomly presented on plates, identified by random 3-digit codes.

### 2.8. Statistical Analysis

Three distinct batches were manufactured for each treatment on different days, and each batch of sausages involved triplicate observations with different sausages. The data were processed using the procedures of Statistix 8.1 (St Paul, MN, USA). The differences between treatments were analyzed using a one-way analysis of variance (ANOVA) and Tukey’s multiple comparison test (*p* < 0.05). The evaluated values follow the normal distribution in analysis by ANOVA. The results are presented as the mean ± standard error (SE). Origin 2023 (Northampton, MA, USA) was used for the principal component analysis (PCA), Spearman rank correlation, and other data plotting. The PCA was carried out on the basis of dimensionality reduction. The sensor response values of the electronic nose and volatile compound content of dry sausage were standardized, respectively, and then their eigenvalues and vectors were calculated, so as to extract the first two principal components and calculate the principal component coefficient. Finally, the connection between the sausages was obtained based on the volatile compound and electronic nose, respectively. A Spearman rank correlation was used to analyze potential correlations between the physicochemical and flavor-quality characteristics. The Spearman rank correlation coefficients were calculated by assigning a rank in order; 1, 0, and −1 represent a positive correlation, no correlation, and negative correlation, respectively.

## 3. Results and Discussion

### 3.1. Analysis of Chemical Composition, pH, and Lactic Acid Bacteria Count

**Figure 1:**
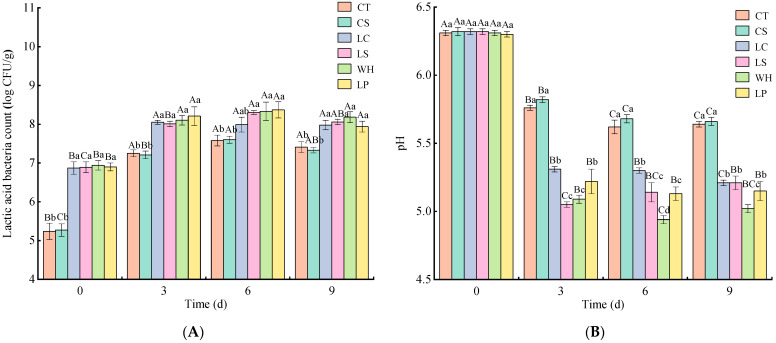

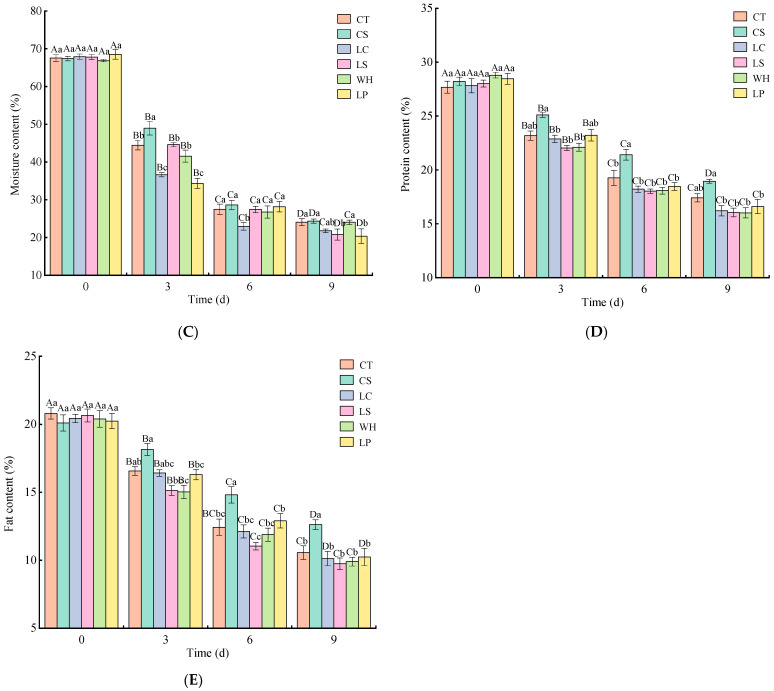
Effects of various lactic acid bacteria on lactic acid bacteria count (**A**), pH (**B**), moisture (**C**), protein (**D**), and fat content (**E**) of dry sausages with NaCl substitutes during fermentation. Different lowercase letters (a–d) denote variations between treatments at the same time, while different uppercase letters (A–D) show variations within same treatments over different times (*p* < 0.05). CT: 100% NaCl; CS: 60% NaCl + 40% KCl; LC: 60% NaCl + 40% KCl + *L. curcatus*; LS: 60% NaCl + 40% KCl + *L. sakei*; WH: 60% NaCl + 40% KCl + *W. hellenica*; LP: 60% NaCl + 40% KCl + *L. plantarum*.

In Figure 1A, the LAB count in the sausages increased consistently during the fermentation process (*p* < 0.05). The LAB counts of the inoculated dry sausages were notably higher than of the uninoculated sausages (*p* < 0.05). On day 3, a rapid growth in LAB was observed in all sausages, suggesting a heightened metabolic activity and growth of LAB during this phase, which aligns with the observed pH results. The LAB count peaked on day 6 for all dry sausages and began to decline by day 9. This decrease can primarily be attributed to the diminishing nutrients and reduced moisture content, both of which restrict LAB growth [24].

As shown in Figure 1B, the pH of the inoculated dry sausages was notably lower than that of the controls (*p* < 0.05). As fermentation proceeded, there was a consistent reduction in the pH of all the sausages, which eventually stabilized or rose slightly by the end of fermentation on day 9. This trend could be attributed to protein degradation, which generates alkaline compounds, and to the slowed metabolic activity of microorganisms [25]. By day 9, the pH of WH was notably lower compared to the others (*p* < 0.05). This may be due to the efficient fermentation abilities of *W. hellenica* [26].

As shown in Figure 1C, the moisture content of all the sausages declined as fermentation continued and stabilized on day 9. This reduction in moisture content is likely due to the drier fermentation environment, leading to ongoing dehydration in the sausages [27]. On day 3, the moisture content in CS was higher than in CT, a phenomenon likely related to the substitution of NaCl with KCl, which can impede water release [11]. Compared to CT and CS, the dry sausages inoculated with NaCl substitutes exhibited a reduced moisture content on day 3. By day 9, LS and LP experienced a significant reduction in moisture content (*p* < 0.05), whereas WH’s moisture content was more aligned with CS (*p* > 0.05). This suggests that introducing LAB may elevate water loss in dry sausages, accelerating the drying process. Nevertheless, by day 9, no significant differences in moisture content between CT and CS were observed, which aligns with the results of Armenteros et al. [28] that replacing NaCl with KCl does not significantly impact the moisture content of the final product.

As shown in Figure 1D, the protein content in all the dry sausages reduced consistently during fermentation. CS had a higher protein content and was notably higher than the others on day 6 of fermentation (*p* < 0.05). There was no obvious difference between the protein content of CT, LC, LS, WH, and LP during fermentation (*p* > 0.05). The fat content of all sausages showed a similar trend to the protein content during fermentation (Figure 1E). The fat content in all the sausages also reduced consistently during fermentation. On days 6 and 9, CS had a higher fat content and was notably higher than the others (*p* < 0.05). The fat content of CT was not significantly different from that of LC, LS, WH, and LP on days 6 and 9 (*p* > 0.05). The above results show that substituting NaCl partially with KCl may reduce the loss of protein and fat. Ions are promoted to release when NaCl is replaced by KCl; the ability of NaCl to release sodium ions (Na^+^) from heme-binding proteins is diminished [29]. Therefore, substituting NaCl partially with KCl may slow lipid oxidation and protein hydrolysis in dry sausage. In addition, inoculation with LAB can promote the lipid and protein hydrolysis of dry sausages with NaCl partly substituted by KCl, mainly related to the effects of lipase and protease in LAB [30].

### 3.2. Color and Shear Force Analysis

The color parameters and shear force of all the dry sausages are listed in Table 1. Throughout the fermentation from day 0 to day 6, the L*-value of all the dry sausages declined, stabilizing by day 9. On day 9, the L*-value of LS and LP were notably lower than that of CS (*p* < 0.05). Inoculation with *L. plantarum* and *L. sakei* can decrease the L*-value of dry sausages with NaCl substitution. This decrease may be related to water loss [31]. From day 0 to day 6 of fermentation, the a*-value for all the dry sausages increased, reaching stability by day 9. The enhancement in pigmentation was due to the formation of nitroso myoglobin in meat, and the reduced moisture content further intensified the color, leading to an increase in a*-value [32]. On day 9, no significant differences in a*-value were noticed between the sausages (*p* > 0.05). On day 9, CS had a higher b*-value compared to CT, LS, and WH, possibly due to the yellow pigment produced by a reaction between the products of lipid oxidation and the amines of phospholipid head groups or proteins [33]. This was followed, in descending order, by LC, LP, WH, CT, and LS (*p* < 0.05). This indicates that substituting NaCl partly with KCl might hasten the lipid oxidation in dry sausages. Notably, inoculation with *L. sakei* significantly decreased the b*-value, largely attributed to its antioxidant properties [34].

Throughout fermentation, the shear force of all the dry sausages increased due to the continuous reduction in moisture content. The shear force of CS was notably lower than that of CT on day 9 (*p* < 0.05). This phenomenon is supported by studies suggesting that potassium ions (K^+^) can react with proteins on the muscle surface. This interaction inhibits the penetration of Na^+^, subsequently reducing the drying rate of dry sausages [35]. Additionally, the shear force of LC was notably higher than that of CS on day 9 (*p* < 0.05). However, there was no significant difference between LC, LS, WH, and CT on day 9 (*p* > 0.05). This indicated that the introduction of *L. sakei*, *L. curvatus*, and *W. hellenica* to sausages where NaCl is partly substituted by KCl can increase their shear force, bringing it on par with the force observed in traditional dry sausages without NaCl substitutes.

### 3.3. Electronic Nose Analysis

**Figure 2:**
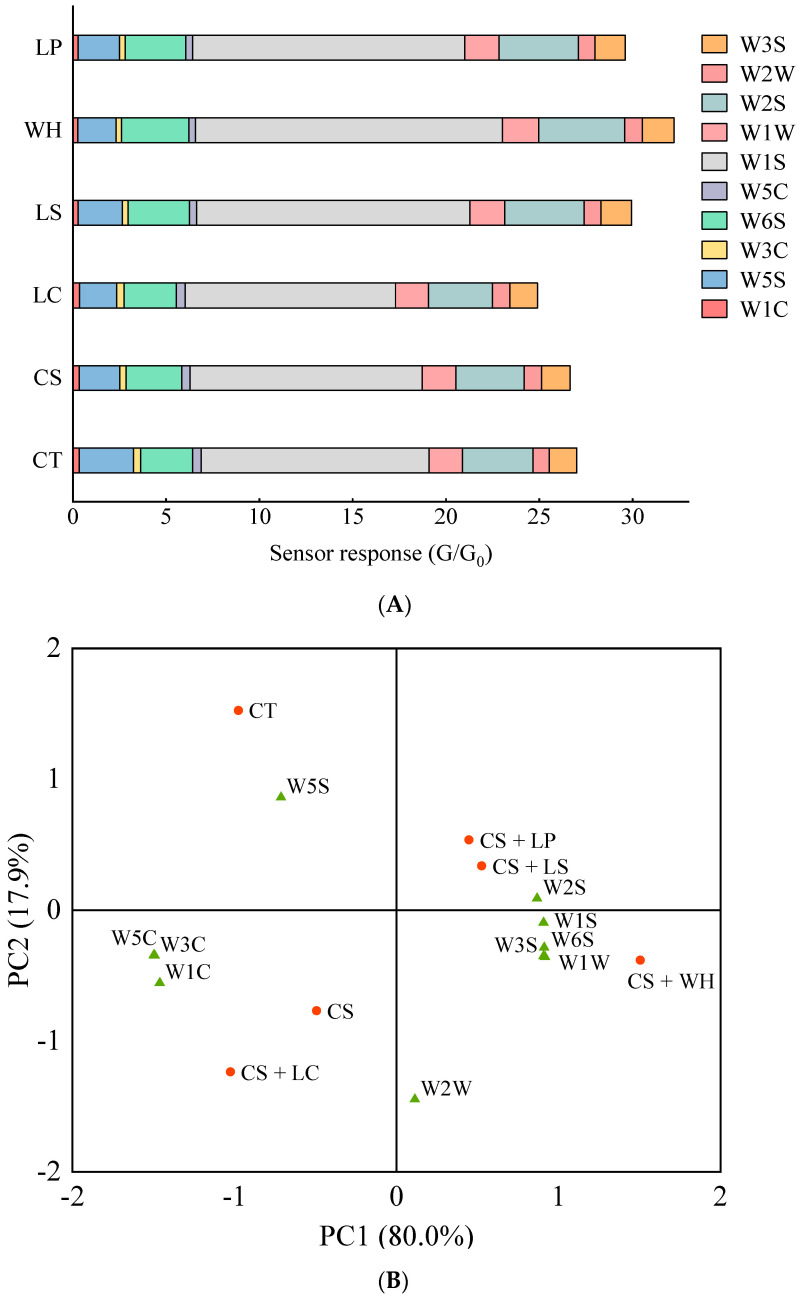
Sensor response (**A**) and principal component analysis (**B**) of the electronic nose for dry sausages with NaCl substitutes inoculated with different lactic acid bacteria on day 9. W1C: aromatic; W5S: broad range; W3C: aromatic; W6S: hydrogen; W5C: arom-aliph; W1S: broad-methane; W1W: sulfur-organic; W2S: broad-alcohol; W2W: sulf-chlor; W3S: methane-aliph. CT: 100% NaCl; CS: 60% NaCl + 40% KCl; LC: 60% NaCl + 40% KCl + *L. curcatus*; LS: 60% NaCl + 40% KCl + *L. sakei*; WH: 60% NaCl + 40% KCl + *W. hellenica*; LP: 60% NaCl + 40% KCl + *L. plantarum*.

An E-nose can quickly analyze the odors of fermented foods, making it a valuable tool for studying the flavor profiles of such foods [18]. As shown in Figure 2A, all treatments had higher response values for sensor W1S, and the sensor responses of LS, WH, and LP were higher than those of CS and CT. Additionally, the responses for W6S and W2S from WH were increased and the responses for W5C and W3C were decreased. These findings suggest that inoculation with *W. hellenica*, *L. sakei*, and *L. plantarum* might enhance the methane component’s formation in dry sausages where NaCl was partially substituted by KCl. Meanwhile, inoculation with *W. hellenica* may also have increased the production of hydrogen and alcohol compounds in dry sausages where NaCl was partially substituted by KCl. As shown in Figure 2B, the explanatory degrees of the first and second components (PC1 and PC2) were 80.0% and 17.9%, totaling explanatory degrees of 97.9%. PC1 emerged as the most significant principal component when distinguishing between sausages. The general flavors of WH, LS, and LP were similar, and these were closely associated with the W2S, W1S, W1W, W6S, and W3S sensors. This suggests that *W. hellenica*, *L. sakei*, and *L. plantarum* may contribute to the production of esters and other aromatic compounds. The overall flavor of CT was strongly associated with the W5S sensor, while the flavors of CS and LC resembled each other, having strong correlations with the W1C, W3C, and W5C sensors.

### 3.4. Volatile Compound Analysis

**Figure 3:**
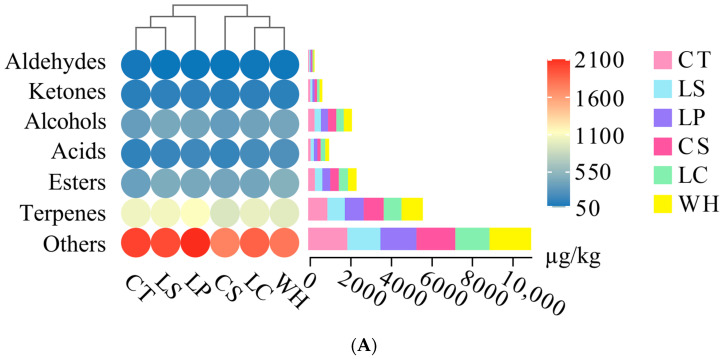

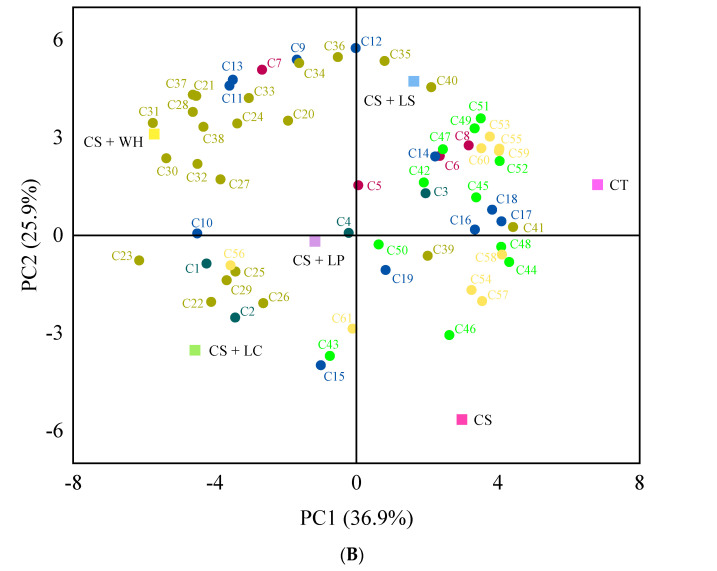
Classification (**A**) and principal component analysis (**B**) of volatile compounds in dry sausages with NaCl substitutes inoculated with various lactic acid bacteria on day 9. CT: 100% NaCl; CS: 60% NaCl + 40% KCl; LC: 60% NaCl + 40% KCl + *L. curcatus*; LS: 60% NaCl + 40% KCl + *L. sakei*; WH: 60% NaCl + 40% KCl + *W. hellenica*; LP: 60% NaCl + 40% KCl + *L. plantarum*.

In Table 2 and Figure 3A, 61 volatile compounds were identified, including 4 aldehydes, 4 ketones, 7 acids, 15 esters, 11 terpenes, 11 alcohols, and 9 other compounds in all sausages. The formation of these compounds is complex, and they are mainly affected by lipid oxidation, protein hydrolysis, microbial metabolism, and flavoring agents [36,37]. Aldehydes are mainly related to protein hydrolysis and lipid oxidation [38]. The aldehydes of LS, LP, and CS were lower than those of CT (*p* < 0.05). The findings suggest that substituting NaCl partially with KCl might decelerate lipid oxidation and protein hydrolysis. This could be attributed to the diminished capacity of NaCl to release Na^+^ from heme-binding proteins when partially replaced by KCl, as NaCl promotes iron liberation [29]. Additionally, inoculation with LAB can enhance lipid and protein hydrolysis in dry sausages, which can be explained by the results of Figure 1D,E—that is, the protein and fat content of LC, LS, WH, and LP were notably lower than those of CS on day 9 (*p* < 0.05). This is associated with the lipase and protease in LAB, which expedite both processes [30]. Aldehydes may considerably influence the flavor of dry sausages. Notably, hexanal, nonanal, and 10-undecenal, primary products of lipid oxidation, are frequently found in fermented meat products [17]. Hexanal emits grassy, tallow, and fatty scents, nonanal exudes waxy, aldehydic, and rose aromas, and 10-undecenal possesses coconut, peach, apricot, and pear fragrances [39].

All of the dry sausages contained four ketones, stemming from lipid oxidation and microbial metabolism [40]. CS had the least ketone content, followed sequentially by CT, LC, WH, LP, and LS (*p* < 0.05). The elevated ketone content in CT compared to CS could be due to the more robust oxidizing properties of Na^+^ over K^+^, while the increased ketone presence in the inoculated dry sausages is potentially due to microbial metabolism [29,30]. Among the ketones, 2-nonanone has rose and tea aromas, 3-hydroxy-2-butanone offers sweet, buttery, and creamy fragrances, sulcatone provides a hazelnut scent, and fenchone has a camphor-like aroma [17]. Specifically, 2-nonanone, 3-hydroxy-2-butanone, and sulcatone likely play a pivotal role in shaping flavor. The content of 3-hydroxy-2-butanone in LS and LP were notably higher than in the others (*p* < 0.05). Apart from adding frankincense and fruity notes to dry sausage, 3-hydroxy-2-butanone also acts as a precursor for synthesizing flavor compounds [41].

Alcohols constituted the main volatile compound in dry sausages, with 11 distinct alcohols identified. CS exhibited the lowest alcohol content, sequentially followed by CT, LC, LP, WH, and LS (*p* < 0.05). Alcohols arise predominantly from lipid oxidation and generally impart a pleasant aroma [1]. Among alcohols, ethanol and 2,3-butanediol can be produced by microbial metabolism. Meanwhile, ethanol may also be related to the addition of *Daqu* during sausage processing. LS exhibited the highest ethanol content, sequentially followed by WH, LP, CS, LC, and CT. While ethanol emits strong ethereal, alcoholic odors, 2,3-butanediol has creamy, fruity, and buttery odors. Ethanol and 2,3-butanediol likely exert minimal influence on the flavor profiles of dry sausages. Similarly, benzyl alcohol, being an aromatic alcohol, has little effect on flavor formation. Conversely, 2-heptanol with green, musty, and leafy aromas; 2-nonanol with waxy, green, and creamy aromas; and linalool with citrus, floral, and sweet aromas potentially play a key role in flavor development [39].

Seven distinct acids were identified in all dry sausages. These primarily derive from phospholipid hydrolysis, lipid oxidation, or microbial metabolism [42]. CT displayed the lowest acid content, followed in order by CS, LS, LP, LC, and WH. Acetic acid, characterized by its sharp, pungent, and sour odor, dominated in content. Acetic acid likely has a limited influence on sausage flavor profiles. In contrast, for example, butanoic acid can significantly influence the flavor of dry sausage, introducing rancid, cheese, and sweat odors [17].

In all the dry sausages, 16 unique esters were identified, and CT registered the lowest ester content, followed sequentially by CS, LC, LP, LS, and WH (*p* < 0.05). Esters may be synthesized from wine or esterification reactions, by microorganisms through acetyl-CoA and higher alcohols, and from a variety of amino acids, reinforcing the essence of ester formation [39,43]. Esters are generally considered to be one of the most important contributors to flavor due to their lower odor threshold and higher content in all volatile compounds [44]. Notably, high contents of bornyl acetate, ethyl acetate, methyl hexanoate, ethyl caprylate, ethyl caprate, methyl butyrate, and ethyl hexanoate were present across all sausages, each contributing various aromatic notes. Methyl butyrate has fruity, apple, and sweet odors. Methyl hexanoate has fruity, pineapple, and ether odors. Ethyl hexanoate has fruity, floral, and sweet odors. Ethyl caprylate has fruity, wine, and waxy odors. Ethyl caprate has waxy, sweet, and fruity odors. Bornyl acetate has a sweet and woody odor. Ethyl acetate has solvent, pineapple, fruity, and apple odors [39]. In addition, volatile compounds derived from spices were also identified, such as β-myrcene and bornyl acetate, while they did not notably differ between treatments (*p* > 0.05).

In Figure 3B, the explanatory degrees of PC1 and PC2 are 36.9% and 25.9%, and the total explanatory degree is 62.8%. CT and LS are closely related to 4-terpinenyl acetate, ethyl hexanoate, and bornyl acetate in the first quadrant. WH is closely related to methyl hexanoate, methyl octanoate, and methyl 3-phenylpropionate in the second quadrant. LC and LP are closely related to hexanoic acid, 3-hydroxybutyric acid, and nonanal in the third quadrant. For the fourth quadrant, CS is closely associated with β-phellandrene, octadecane, and eugenol. The above provides a valuable understanding of the variation in volatile compounds in the sausages.

### 3.5. Sensory Evaluation

**Figure 4:**
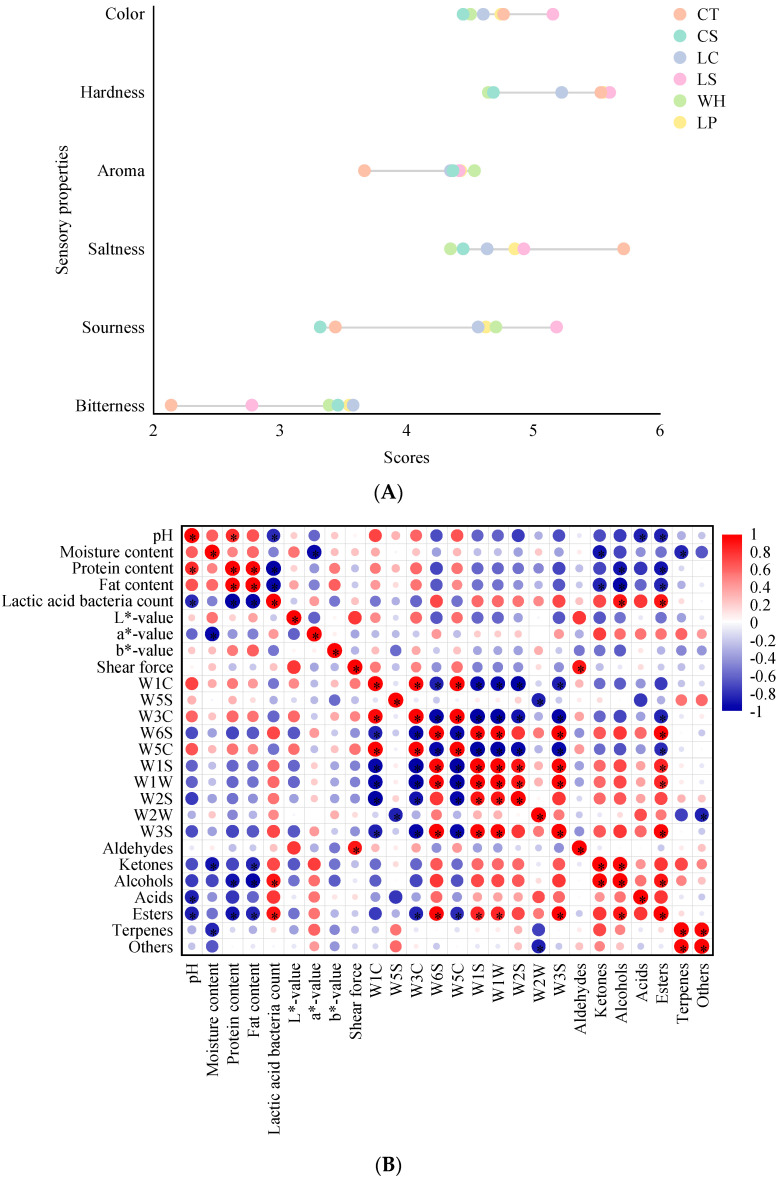
Sensory evaluation (**A**) and Spearman rank correlation between physicochemical characteristics, flavor, and quality (**B**) of dry sausages with NaCl substitutes inoculated with various lactic acid bacteria. W1C: aromatic; W5S: broad range; W3C: aromatic; W6S: hydrogen; W5C: arom-aliph; W1S: broad-methane; W1W: sulfur-organic; W2S: broad-alcohol; W2W: sulf-chlor; W3S: methane-aliph. The color scale denotes the nature of the correlation, with 1 indicating a positive correlation (red) and −1 indicating a negative correlation (blue). “*” represents significance at *p* < 0.05. CT: 100% NaCl; CS: 60% NaCl + 40% KCl; LC: 60% NaCl + 40% KCl + *L. curcatus*; LS: 60% NaCl + 40% KCl + *L. sakei*; WH: 60% NaCl + 40% KCl + *W. hellenica*; LP: 60% NaCl + 40% KCl + *L. plantarum*.

As shown in Figure 4A, the color difference between all the dry sausages was not significant on day 9 (*p* > 0.05). The hardness of CS was notably lower than that of CT (*p* < 0.05). However, the hardness of the dry sausages improved with inoculation with *L. curcatus*, *L. sakei*, and *L. plantarum* compared to CS, making it comparable to CT. This finding aligns with the shear force results, mainly attributed to the significant decrease in moisture content in the dry sausages [45]. The aroma of inoculation with LAB was stronger than that of CT, especially in those inoculated with *W. hellenica* and *L. plantarum* (*p* < 0.05). This observation is consistent with the GC-MS results, mainly due to the increased content of esters. The saltiness of CT was notably higher than that of the others, and the sourness of the inoculated dry sausages was notably higher than that of the controls (*p* < 0.05), mainly related to LAB metabolism. The sourness comes mainly from lactic acid, and lactic acid is detected in the highest content in the sausages [46]. Notably, inoculating with *L. sakei* reduced the bitterness in dry sausages where NaCl was partially replaced by KCl, possibly because of the pronounced sour and aromatic flavors in the dry sausages.

### 3.6. Correlation between Physicochemical Characteristics and Flavor Quality

As shown in Figure 4B, esters were significantly negatively correlated with pH, protein, fat content, W3C, and W5C and significantly positively correlated with LAB count, W6S, W1S, W1W, and W3S. Given that esters are key volatile compounds in fermented meat products, they displayed the most significant correlations in this study. Inoculation with LAB potentially boosts ester content by promoting esterification [39,43]. A significant negative correlation was observed between pH and both LAB count and acids. As fermentation continued, the pH of all the dry sausages decreased, and those inoculated with LAB demonstrated enhanced LAB counts and acid content compared to the controls. The moisture content was significantly negatively correlated with a*-value, ketones, and terpenes. Throughout fermentation, there was a consistent reduction in moisture content in all the dry sausages, with a concomitant rise in a*-value, attributed to pigmentation and moisture loss. Alcohols were notably positively correlated with LAB count and notably negatively correlated with protein and fat content, suggesting that alcohols can be formed through LAB metabolism and lipid oxidation and protein hydrolysis [43]. Meanwhile, a notable negative correlation was found between LAB count and both protein and fat content. A notable negative correlation was found between fat and ketone content. The lipase and protease produced by LAB can promote lipid and protein hydrolysis in dry sausage [30]. Lipid oxidation is the main way to produce volatile compounds in the fermentation process, and a variety of volatile compounds are conducive to the formation of characteristic flavors. Fat content was notably positively correlated with protein content. Protein and lipid oxidation have the same catalyst; they can be carried out individually, or they can interact with each other [47].

## 4. Conclusions

Inoculation with LAB (especially *L. sakei*) can improve the color (mainly b*-value) and texture of dry sausages that have partially substituted NaCl with KCl. It also increases acids, alcohols, ketones, and esters, while reducing bitterness. The Spearman rank correlation further confirmed that pH, moisture, protein, fat content, and LAB count play a notable effect in the color and flavor of dry sausage with NaCl substitution. Although a correlation analysis is helpful to identify potential associations, the mechanisms underlying the contribution of LAB inoculation to the flavor and quality of dry sausages still require further investigation. Meanwhile, optimizing inoculation with LAB should be considered, to further improve the product characteristics of dry sausages. In summary, this study provides a strategy and application prospects for improving the flavor and quality characteristics of low-sodium dry sausages.

## Figures and Tables

**Table 1:** Effects of various lactic acid bacteria on the L*, a*, and b*-value and shear force of dry sausages with NaCl substitutes during fermentation.

	Time (d)	CT	CS	LC	LS	WH	LP
L*-value	0	44.96 ± 0.99 ^Aa^	45.81 ± 2.04 ^Aa^	45.23 ± 2.19 ^Aa^	44.73 ± 0.54 ^Aa^	45.17 ± 0.96 ^Aa^	46.19 ± 1.20 ^Aa^
	3	39.90 ± 3.18 ^Bb^	39.65 ± 0.81 ^Bb^	37.69 ± 1.17 ^Bb^	44.23 ± 2.07 ^Aa^	43.47 ± 0.48 ^Ba^	39.13 ± 1.38 ^Bb^
	6	36.62 ± 1.35 ^Cbc^	38.01 ± 1.26 ^BCb^	35.75 ± 0.51 ^Cc^	37.06 ± 1.08 ^Bbc^	40.81 ± 0.78 ^Ca^	34.16 ± 1.05 ^Cd^
	9	38.38 ± 0.81 ^BCa^	37.00 ± 0.69 ^Cb^	37.41 ± 0.60 ^BCab^	33.78 ± 1.05 ^Cd^	37.17 ± 0.66 ^Db^	35.21 ± 1.08 ^Cc^
a*-value	0	12.42 ± 1.62 ^Ca^	11.68 ± 0.81 ^Da^	11.92 ± 0.60 ^Ca^	11.88 ± 1.17 ^Ca^	12.55 ± 0.93 ^Ca^	12.01 ± 0.90 ^Da^
	3	13.67 ± 1.11 ^BCbc^	14.03 ± 0.66 ^Cb^	15.72 ± 0.96 ^Ba^	12.57 ± 1.29 ^Cc^	14.12 ± 0.48 ^Bb^	13.66 ± 1.44 ^Cbc^
	6	14.77 ± 1.20 ^ABd^	17.36 ± 0.81 ^Aab^	16.91 ± 0.96 ^Aab^	18.10 ± 1.02 ^Aa^	16.15 ± 0.69 ^Abc^	15.11 ± 0.30 ^Bcd^
	9	16.13 ± 1.02 ^Aa^	16.23 ± 0.84 ^Ba^	16.53 ± 0.54 ^ABa^	16.53 ± 0.72 ^Ba^	16.25 ± 0.57 ^Aa^	16.59 ± 1.38 ^Aa^
b*-value	0	16.70 ± 1.56 ^Aa^	17.90 ± 0.75 ^Aa^	17.35 ± 0.99 ^Aa^	17.70 ± 0.93 ^Aa^	17.15 ± 1.08 ^Aa^	17.12 ± 0.48 ^Aa^
	3	12.20 ± 1.05 ^Cb^	15.01 ± 0.54 ^Ba^	14.17 ± 0.75 ^Ca^	11.19 ± 0.90 ^Cb^	13.95 ± 1.11 ^Ca^	11.95 ± 0.84 ^Cb^
	6	13.02 ± 0.51 ^Cb^	15.39 ± 1.74 ^Ba^	14.83 ± 0.93 ^BCa^	14.55 ± 0.33 ^Ba^	15.06 ± 0.81 ^BCa^	10.97 ± 0.81 ^Dc^
	9	14.63 ± 0.42 ^Bc^	16.08 ± 0.42 ^Ba^	15.70 ± 0.39 ^Bab^	13.78 ± 0.36 ^Bd^	15.41 ± 0.33 ^Bb^	15.70 ± 0.39 ^Bab^
Shear force (N)	0	2.03 ± 0.17 ^Da^	2.36 ± 0.17 ^Ca^	2.23 ± 0.24 ^Da^	2.32 ± 0.28 ^Da^	2.36 ± 0.28 ^Ca^	2.20 ± 0.14 ^Ca^
	3	8.52 ± 1.25 ^Cab^	7.56 ± 0.43 ^Bab^	7.58 ± 1.30 ^Cab^	5.84 ± 0.62 ^Cb^	9.72 ± 1.20 ^Ba^	8.63 ± 1.21 ^Bab^
	6	11.65 ± 1.06 ^Ba^	11.46 ± 0.78 ^Aa^	12.17 ± 1.07 ^Ba^	9.48 ± 1.35 ^Ba^	11.29 ± 0.73 ^Ba^	11.79 ± 1.84 ^ABa^
	9	16.10 ± 1.06 ^Aa^	12.58 ± 1.28 ^Abc^	16.12 ± 0.52 ^Aa^	14.68 ± 1.04 ^Aabc^	15.32 ± 1.28 ^Aab^	12.08 ± 1.37 ^Ac^

Different lowercase letters (a–d) indicate the difference between different treatments at the same time, and different uppercase letters (A–D) indicate the difference between the same treatment at different times (*p* < 0.05). CT: 100% NaCl; CS: 60% NaCl + 40% KCl; LC: 60% NaCl + 40% KCl + *L. curcatus*; LS: 60% NaCl + 40% KCl + *L. sakei*; WH: 60% NaCl + 40% KCl + *W. hellenica*; LP: 60% NaCl + 40% KCl + *L. plantarum*.

**Table 2:** Effects of various lactic acid bacteria on content (µg/kg) of volatile compounds in dry sausages with NaCl substitutes on day 9.

Number	Volatile Compound	CT	CS	LC	LS	WH	LP
	Aldehydes						
C1	Hexanal	6.26 ± 0.35 ^a^	5.73 ± 0.31 ^ab^	5.13 ± 0.26 ^bc^	5.79 ± 0.36 ^ab^	6.08 ± 0.38 ^a^	4.38 ± 0.12 ^c^
C2	Nonanal	28.56 ± 1.40 ^a^	24.55 ± 1.30 ^ab^	27.90 ± 2.60 ^a^	21.93 ± 1.85 ^b^	26.68 ± 1.28 ^a^	21.72 ± 1.11 ^b^
C3	Cinnamaldehyde	11.76 ± 0.94 ^b^	13.65 ± 0.90 ^ab^	11.46 ± 1.44 ^b^	13.48 ± 0.76 ^ab^	11.07 ± 1.04 ^b^	14.72 ± 0.90 ^a^
C4	10-Undecenal	12.32 ± 1.21 ^a^	4.06 ± 0.21 ^c^	11.22 ± 1.11 ^a^	7.82 ± 0.80 ^b^	10.32 ± 0.89 ^a^	7.72 ± 0.69 ^b^
	Total	58.90 ± 3.90 ^a^	47.99 ± 2.72 ^b^	55.71 ± 5.41 ^a^	49.02 ± 3.77 ^b^	54.15 ± 3.59 ^a^	48.54 ± 2.82 ^b^
	Ketones						
C5	2-Nonanone	3.35 ± 0.35 ^b^	3.82 ± 0.17 ^b^	4.65 ± 0.35 ^a^	3.98 ± 0.31 ^ab^	3.26 ± 0.14 ^b^	4.03 ± 0.31 ^ab^
C6	3-hydroxy-2-butanone	21.34 ± 1.33 ^b^	20.35 ± 1.30 ^b^	16.28 ± 0.68 ^c^	24.66 ± 1.35 ^a^	20.24 ± 0.88 ^b^	24.88 ± 1.39 ^a^
C7	Sulcatone	10.26 ± 0.43 ^c^	10.46 ± 0.38 ^c^	18.36 ± 0.31 ^b^	18.69 ± 0.66 ^b^	25.65 ± 1.11 ^a^	17.38 ± 1.06 ^b^
C8	Fenchone	72.48 ± 3.97 ^ab^	69.47 ± 4.26 ^ab^	75.27 ± 4.31 ^a^	76.89 ± 3.43 ^a^	69.41 ± 4.90 ^ab^	75.90 ± 4.80 ^a^
	Total	107.43 ± 6.08 ^c^	104.10 ± 6.11 ^d^	114.56 ± 5.65 ^b^	124.22 ± 5.75 ^a^	118.56 ± 7.03 ^ab^	122.19 ± 7.56 ^a^
	Alcohols						
C9	Ethanol	106.87 ± 7.90 ^e^	140.04 ± 7.40 ^c^	126.80 ± 5.06 ^d^	188.41 ± 10.50 ^a^	162.43 ± 10.27 ^b^	150.37 ± 9.72 ^c^
C10	2,3-Butanediol	14.70 ± 1.00 ^c^	9.15 ± 0.62 ^d^	19.73 ± 1.18 ^b^	4.44 ± 0.12 ^f^	23.95 ± 0.57 ^a^	6.48 ± 0.29 ^e^
C11	2-Ethylhexanol	12.10 ± 0.28 ^b^	11.91 ± 0.40 ^b^	12.74 ± 0.23 ^ab^	13.56 ± 0.17 ^a^	13.83 ± 0.21 ^a^	11.94 ± 0.31 ^b^
C12	2-Heptanol	n.d.	n.d.	2.05 ± 0.21 ^b^	3.19 ± 0.19 ^a^	2.35 ± 0.05 ^b^	2.48 ± 0.23 ^b^
C13	2-Nonanol	n.d.	n.d.	18.39 ± 1.02 ^b^	18.66 ± 1.21 ^b^	25.63 ± 1.28 ^a^	4.58 ± 0.19 ^c^
C14	Benzyl alcohol	8.60 ± 0.31 ^c^	9.34 ± 0.42 ^c^	11.93 ± 0.97 ^b^	9.94 ± 0.73 ^c^	9.73 ± 0.38 ^c^	15.37 ± 1.13 ^a^
C15	Phenethyl alcohol	19.53 ± 1.54 ^a^	19.64 ± 1.06 ^a^	20.18 ± 0.80 ^a^	15.31 ± 0.90 ^b^	15.38 ± 0.73 ^b^	17.45 ± 1.16 ^ab^
C16	3-phenylpropanol	22.61 ± 0.50 ^c^	2.82 ± 0.29 ^e^	27.52 ± 1.30 ^a^	17.15 ± 0.83 ^d^	2.83 ± 0.17 ^e^	24.77 ± 0.68 ^b^
C17	Terpinen-4-ol	60.67 ± 3.34 ^ab^	52.53 ± 2.03 ^b^	59.56 ± 4.07 ^ab^	57.81 ± 2.18 ^ab^	54.21 ± 4.26 ^ab^	62.55 ± 3.57 ^a^
C18	α-Terpineol	32.62 ± 1.97 ^ab^	25.14 ± 1.51 ^c^	28.55 ± 1.66 ^bc^	29.07 ± 2.03 ^bc^	28.88 ± 1.11 ^bc^	35.37 ±1.73 ^a^
C19	Linalool	42.34 ± 3.19 ^ab^	43.66 ± 1.11 ^a^	35.46 ± 2.23 ^b^	40.41 ± 2.46 ^ab^	39.50 ± 1.47 ^ab^	43.92 ± 1.96 ^a^
	Total	320.04 ± 20.03 ^d^	314.23 ± 14.84 ^e^	362.91 ± 18.73 ^c^	397.95 ± 21.32 ^a^	378.72 ± 20.50 ^b^	375.28 ± 20.97 ^b^
	Acids						
C20	Acetic acid	84.72 ± 7.01 ^cd^	71.23 ± 6.50 ^d^	124.61 ± 8.04 ^b^	90.13 ± 5.70 ^c^	146.31 ± 8.00 ^a^	119.47 ± 5.99 ^b^
C21	Butanoic acid	4.65 ± 0.21 ^e^	12.06 ± 0.57 ^d^	17.23 ± 0.71 ^b^	14.21 ± 0.80 ^c^	19.17 ± 1.04 ^a^	10.85 ± 0.54 ^d^
C22	Hexanoic acid	9.98 ± 0.23 ^a^	7.60 ± 0.16 ^b^	3.62 ± 0.21 ^c^	3.59 ± 0.12 ^c^	10.00 ± 0.40 ^a^	2.96 ± 0.16 ^d^
C23	Heptanoic acid	15.16 ± 1.45 ^c^	19.46 ± 0.66 ^a^	18.83 ± 0.50 ^ab^	15.47 ± 0.48 ^c^	17.12 ± 0.42 ^bc^	11.98 ± 0.31 ^d^
C24	Octanoic acid	2.12 ± 0.07 ^d^	23.45 ± 1.28 ^b^	27.46 ± 1.45 ^a^	17.24 ± 1.70 ^c^	26.16 ± 1.65 ^ab^	24.52 ± 1.21 ^ab^
C25	Nonanoic acid	8.31 ± 0.24 ^a^	5.46 ± 0.23 ^c^	8.00 ± 0.53 ^a^	6.50 ± 0.40 ^b^	6.57 ± 0.31 ^b^	2.42 ± 0.10 ^d^
C26	3-Hydroxybutyric acid	4.69 ± 0.12 ^a^	4.67 ± 0.16 ^a^	n.d.	n.d.	4.98 ± 0.07 ^a^	3.15 ± 0.12 ^b^
	Total	129.63 ± 9.33 ^e^	143.93 ± 9.56 ^d^	199.75 ± 11.44 ^b^	147.14 ± 9.20 ^d^	230.31 ± 11.89 ^a^	175.35 ± 8.43 ^c^
	Esters						
C27	Methyl acetate	n.d.	4.56 ± 0.43 ^a^	n.d.	2.45 ± 0.09 ^bc^	2.89 ± 0.12 ^b^	2.02 ± 0.19 ^c^
C28	Methyl lactate	n.d.	n.d.	2.30 ± 0.10 ^b^	2.23 ± 0.07 ^b^	6.54 ± 0.42 ^a^	n.d.
C29	Methyl butyrate	12.32 ± 1.18 ^b^	13.88 ± 1.13 ^b^	16.28 ± 0.57 ^a^	2.23 ± 0.07 ^c^	17.34 ± 0.68 ^a^	12.50 ± 0.33 ^b^
C30	Methyl hexanoate	45.85 ± 2.13 ^d^	73.91 ± 3.93 ^b^	59.97 ± 3.15 ^c^	55.37 ± 2.81 ^cd^	87.15 ± 6.08 ^a^	62.33 ± 5.84 ^c^
C31	Methyl octanoate	n.d.	3.83 ± 0.17 ^c^	2.40 ± 0.09 ^d^	4.61 ± 0.29 ^b^	5.63 ± 0.31 ^a^	n.d.
C32	Methyl 3-phenylpropionate	4.70 ± 0.16 ^d^	9.01 ± 0.45 ^c^	12.20 ± 0.94 ^b^	4.64 ± 0.14 ^d^	16.50 ± 1.75 ^a^	9.63 ± 0.66 ^c^
C33	Ethyl acetate	30.41 ± 1.14 ^d^	38.16 ± 2.79 ^c^	39.48 ± 2.58 ^c^	71.70 ± 4.33 ^a^	51.31 ± 2.25 ^b^	25.85 ± 1.11 ^d^
C34	Ethyl butyrate	4.53 ± 0.17 ^d^	4.72 ± 0.21 ^d^	7.96 ± 0.26 ^b^	10.12 ± 0.61 ^a^	7.82 ± 0.61 ^b^	6.06 ± 0.59 ^c^
C35	Ethyl hexanoate	67.80 ± 6.48 ^b^	60.75 ± 5.30 ^b^	70.33 ± 7.01 ^b^	94.06 ± 5.98 ^a^	88.22 ± 5.18 ^a^	90.83 ± 5.80 ^a^
C36	Ethyl heptanoate	3.39 ± 0.40 ^b^	3.09 ± 0.09 ^b^	4.39 ± 0.17 ^a^	4.72 ± 0.21 ^a^	5.00 ± 0.26 ^a^	4.69 ± 0.33 ^a^
C37	Ethyl caprylate	10.13 ± 0.88 ^c^	13.89 ± 0.74 ^b^	13.06 ± 0.73 ^b^	14.00 ± 0.99 ^b^	17.92 ± 1.11 ^a^	13.67 ± 0.61 ^b^
C38	Ethyl caprate	7.40 ± 0.33 ^c^	9.35 ± 0.50 ^bc^	9.43 ± 0.43 ^b^	8.63 ± 0.59 ^bc^	14.24 ± 1.16 ^a^	10.03 ± 0.97 ^b^
C39	γ-Butyrolactone	7.72 ± 0.40 ^b^	9.30 ± 0.10 ^a^	4.75 ± 0.43 ^c^	8.65 ± 0.64 ^ab^	3.73 ± 0.28 ^c^	9.33 ± 0.36 ^a^
C40	4-Terpinenyl acetate	3.32 ± 0.21 ^b^	3.45 ± 0.16 ^ab^	3.69 ± 0.23 ^ab^	3.98 ± 0.35 ^ab^	3.56 ± 0.33 ^ab^	4.13 ± 0.40 ^a^
C41	Bornyl acetate	142.18 ± 7.98 ^a^	131.47 ± 10.08 ^a^	134.47 ± 5.66 ^a^	137.36 ± 6.46 ^a^	132.00 ± 8.00 ^a^	147.51 ± 7.88 ^a^
	Total	339.75 ± 21.46 ^e^	379.37 ± 26.08 ^d^	380.71 ± 22.35 ^d^	424.75 ± 23.63 ^b^	459.85 ± 28.54 ^a^	398.58 ± 25.07 ^c^
	Terpenes						
C42	α-Cubebene	18.94 ± 1.42 ^a^	15.07 ± 0.68 ^b^	17.77 ± 0.83 ^ab^	17.32 ± 2.08 ^ab^	18.75 ± 0.73 ^a^	19.23 ± 1.77 ^a^
C43	α-Muurolene	21.41 ± 0.94 ^ab^	23.27 ± 1.97 ^a^	19.08 ± 1.00 ^b^	17.46 ± 2.04 ^b^	18.51 ± 1.73 ^b^	21.30 ± 1.33 ^ab^
C44	α-Curcumene	98.57 ± 6.50 ^a^	83.37 ± 16.71 ^a^	88.34 ± 4.52 ^a^	89.88 ± 7.27 ^a^	82.30 ± 10.25 ^a^	98.68 ± 7.15 ^a^
C45	β-Caryophyllene	167.83 ± 7.67 ^a^	125.27 ± 5.06 ^a^	151.95 ± 7.79 ^b^	152.49 ± 11.71 ^a^	151.80 ± 8.23 ^a^	171.79 ± 9.01 ^a^
C46	β-Phellandrene	28.45 ± 1.37 ^a^	23.28 ± 2.51 ^b^	24.94 ± 1.30 ^ab^	21.80 ± 1.70 ^b^	21.99 ± 1.68 ^b^	26.65 ± 2.39 ^ab^
C47	β-Ocimene	4.27 ± 0.12 ^bc^	4.00 ± 0.17 ^c^	4.70 ± 0.21 ^b^	4.36 ± 0.24 ^bc^	4.59 ± 0.35 ^bc^	5.67 ± 0.36 ^a^
C48	β-Muurolene	38.29 ± 2.56 ^a^	29.20 ± 1.28 ^d^	33.27 ± 1.40 ^bcd^	34.16 ± 1.61 ^abc^	30.68 ± 1.52 ^cd^	37.22 ± 2.04 ^ab^
C49	β-Myrcene	52.51 ± 3.60 ^a^	51.65 ± 2.81 ^a^	52.50 ± 2.48 ^a^	58.44 ± 3.62 ^a^	50.75 ± 2.23 ^a^	58.44 ± 2.08 ^a^
C50	Camphene	5.93 ± 0.21 ^b^	4.14 ± 0.07 ^c^	7.07 ± 0.16 ^a^	5.79 ± 0.26 ^b^	3.64 ± 0.12 ^d^	4.45 ± 0.10 ^c^
C51	D-Limonene	453.40 ± 19.66 ^ab^	432.63 ± 12.45 ^b^	455.85 ± 25.60 ^ab^	503.47 ± 41.85 ^ab^	442.96 ± 18.69 ^ab^	511.86 ± 31.80 ^a^
C52	γ-Terpinene	75.45 ± 7.00 ^ab^	62.53 ± 5.68 ^b^	70.30 ± 4.19 ^ab^	77.70 ± 4.28 ^a^	68.17 ± 3.05 ^ab^	79.53 ± 4.85 ^a^
	Total	965.05 ± 51.05 ^b^	854.41 ± 49.39 ^e^	925.77 ± 49.48 ^c^	982.87 ± 76.66 ^b^	894.14 ± 48.58 ^d^	1034.82 ± 62.88 ^a^
	Others						
C53	D-Camphor	227.93 ± 10.36 ^bc^	203.23 ± 14.72 ^c^	227.97 ± 12.12 ^bc^	240.98 ± 14.24 ^ab^	222.69 ± 8.78 ^bc^	266.82 ± 7.98 ^a^
C54	Octadecane	58.14 ± 2.63 ^a^	46.03 ± 3.00 ^c^	51.31 ± 2.04 ^abc^	47.89 ± 2.88 ^bc^	48.09 ± 2.36 ^bc^	55.71 ± 3.59 ^ab^
C55	P-Cymene	23.40 ± 1.77 ^ab^	20.39 ± 0.54 ^b^	21.81 ± 1.77 ^ab^	24.84 ± 2.15 ^a^	21.47 ± 1.44 ^ab^	25.30 ± 1.54 ^a^
C56	Safrole	56.49 ± 3.12 ^b^	63.35 ± 3.22 ^b^	81.08 ± 4.31 ^a^	44.71 ± 2.72 ^c^	63.01 ± 2.59 ^b^	56.16 ± 3.95 ^b^
C57	Eugenol	495.10 ± 22.71 ^a^	419.62 ± 16.21 ^b^	428.27 ± 22.53 ^b^	407.10 ± 12.12 ^b^	399.21 ± 15.64 ^b^	501.71 ± 26.33 ^a^
C58	4-Allylanisole	106.83 ± 6.63 ^ab^	79.72 ± 4.52 ^c^	90.31 ± 5.23 ^bc^	89.67 ± 4.50 ^bc^	83.76 ± 6.51 ^c^	109.06 ± 11.02 ^a^
C59	1,8-Cineole	114.17 ± 5.13 ^ab^	107.60 ± 6.20 ^b^	115.89 ± 9.02 ^ab^	121.35 ± 9.11 ^ab^	108.58 ± 6.30 ^b^	128.99 ± 8.61 ^a^
C60	Anethole	804.94 ± 27.85 ^b^	654.40 ± 15.17 ^d^	724.48 ± 18.83 ^c^	892.66 ± 25.84 ^a^	704.53 ± 31.54 ^cd^	841.78 ± 29.06 ^ab^
C61	Methyl eugenol	47.72 ± 2.44 ^a^	41.29 ± 2.18 ^ab^	48.47 ± 3.59 ^a^	33.39 ± 2.60 ^b^	41.16 ± 4.02 ^ab^	45.01 ± 2.27 ^a^
	Total	1934.72 ± 82.64 ^b^	1635.63 ± 65.76 ^d^	1789.59 ± 79.44 ^c^	1902.59 ± 76.16 ^b^	1692.50 ± 79.18 ^d^	2030.54 ± 94.35 ^a^

Different lowercase letters (a–e) indicate significant differences between dry sausages (*p* < 0.05). n.d.: volatile compounds not detected. CT: 100% NaCl; CS: 60% NaCl + 40% KCl; LC: 60% NaCl + 40% KCl + *L. curcatus*; LS: 60% NaCl + 40% KCl + *L. sakei*; WH: 60% NaCl + 40% KCl + *W. hellenica*; LP: 60% NaCl + 40% KCl + *L. plantarum*.

## Data Availability

The original contributions presented in the study are included in the article, further inquiries can be directed to the corresponding authors.
